# The Prevalence and Risk Factors of Paternal Depression from the Antenatal to the Postpartum Period and the Relationships between Antenatal and Postpartum Depression among Fathers in Hong Kong

**DOI:** 10.1155/2014/127632

**Published:** 2014-01-28

**Authors:** Y. W. Koh, C. Y. Chui, C. S. K. Tang, A. M. Lee

**Affiliations:** ^1^The University of Hong Kong, Hong Kong; ^2^National University of Singapore, Singapore; ^3^Department of Psychiatry, The University of Hong Kong, Pokfulam, Hong Kong

## Abstract

*Introduction*. Despite the fact that maternal perinatal mental health problems have been extensively studied and addressed to be a significant health problem, the literature on paternal perinatal mental health problems is relatively scarce. The present study aims at determining the prevalence of paternal perinatal depression and identifying the risk factors and the relationship between antenatal and postpartum depression. *Methodology*. 622 expectant fathers were recruited from regional maternal clinics. The expectant fathers were assessed using standardized and validated psychological instruments on 3 time points including early pregnancy, late pregnancy, and six weeks postpartum. *Results*. Results showed that a significant proportion of expectant fathers manifested depressive symptoms during the perinatal period. Paternal antenatal depression could significantly predict higher level of paternal postpartum depression. Psychosocial risk factors were consistently associated with paternal depression in different time points. *Conclusions*. The present study points to the need for greater research and clinical attention to paternal depression given that it is a highly prevalent problem and could be detrimental to their spouse and children development. The present findings contribute to theoretical basis of the prevalence and risk factors of paternal perinatal depression and have implications of the design of effective identification, prevention, and interventions of these clinical problems.

## 1. Introduction

Maternal perinatal mental health problems have been extensively studied and addressed to be a significant health problem. Postpartum depression, in particular, has been thoroughly studied and recognized to be a significant mental health problem with a prevalence of 10–20% among women [1–5]. In comparison, paternal mental health problems are largely underresearched. However, there is emerging evidence of the significance of mental health problems among the fathers in the postpartum period. Reported rates of postpartum depression among fathers ranged from 5.3 to 31.7% [6–9]. A meta-analysis of 43 studies showed that antenatal and postpartum depression was evident in about 10% of men [10]. It is widely recognized that paternal mental ill health could increase the risk of behavioral and emotional problems in children [11, 12]. Most of the previous studies mainly focused on expectant fathers' postpartum depression. Nevertheless, a pioneer study on first time fathers clearly demonstrated that pregnancy is a more stressful period for most men than the postpartum period [13]. Preliminary findings showed that depression affected from 4.8 to 12% of fathers in the antenatal period [6, 14]. To date, there is very limited knowledge on paternal depression during the antenatal period, and even less studies investigating it in a longitudinal manner, particularly examining its relationship with paternal postpartum depression. If paternal antenatal depression is a reliable predictor of paternal postpartum depression, screening and intervention could be instituted much early on to prevent mental ill health in the postpartum period.

Studies on the relationship between maternal antenatal and postpartum depression showed that maternal depression during pregnancy was the strongest predictor of maternal postpartum depression [3, 15, 16]. However, the relationship between paternal antenatal and postpartum depression remains largely unknown. Knowledge on risk factors of paternal depression is also scarce. Preliminary studies showed that some risk factors associated with paternal antenatal psychological distress included poor marital relationship, poor social network, and insufficient information about pregnancy and childbirth [17]. On the other hand, poor marital satisfaction, low social support and poor quality of life were found to be associated with paternal postpartum depression [17]. Having an unsupportive relationship, marital disharmony, being unemployed, young age, poorer social functioning and past history of psychiatric disorder were also found to be associated with paternal mental health problems during the perinatal period [7, 18–20]. Matthey et al. [21] stated that adjustment to parenthood was related to different variables at different times of the perinatal period [21]. It is therefore important to examine risk factors using a longitudinal design. However, to the best of our knowledge, there is no longitudinal study in the existing literature specifically on risk factors identification with respect to paternal depression. By identifying risk factors and their relationship with paternal depression in a longitudinal manner, the study would be able to render findings that would impact our conceptual understanding of the difficulties faced by expectant fathers as well as aid in the development of clinical preventive strategies to improve paternal well-being.

Thus, the present study aims at examining the prevalence of paternal depression in early, late pregnancy, and six weeks postpartum, investigating the relationship between paternal antenatal and postpartum depression and identifying risk factors among expectant fathers in a longitudinal manner.

## 2. Methodology

A consecutive sample of 622 expectant fathers was recruited from the antenatal clinics in two major regional hospitals in Hong Kong. The inclusion criteria included (1) expectant fathers, (2) fathers of Chinese ethnicity, and (3) fathers being able to read and write Chinese. The exclusion criteria included (1) couples considering termination of pregnancy (2) fathers with primary residence outside of Hong Kong. The expectant fathers were administered a set of questionnaires at first presentation (12 weeks gestation of pregnancy) in the antenatal clinic at a regional hospital in Hong Kong. The questionnaires were completed the expectant fathers during the waiting time in the antenatal clinic. They were re-assessed when the pregnancy progressed to 36 weeks and again at 6 weeks after childbirth. In all, the participants were assessed on 3 time-points. The study was approved by the Institutional Review Board of the university and the hospital. All eligible expectant fathers attending the antenatal clinic at first antenatal presentation were invited to participate in the study. Subjects were informed about the objective, background, and procedure of the research. Upon providing informed written consent, subjects completed a set of self-administered questionnaire.

### 2.1. Measurement of Paternal Depression

The Edinburgh Postnatal Depression Scale (EPDS) [22] was used to assess depressive symptoms in the antenatal and postpartum periods. It is a self-report measure consisting of 10 items and each item is rated on a four-point scale. It is a well-validated and the most widely used screening measure of postpartum depression among women. It has also been validated for use in the antenatal period [23] and among men as measure of paternal depression [9]. The Chinese version of the EPDS has been validated among pregnant women with satisfactory psychometric properties [24]. The recommended cut-off of 12/13 was used to define a probable case of depression. Cronbach's alpha for the EPDS is .87 [22].

### 2.2. Measurements of Risk Factors

Demographic risk factors including age, educational level, marital status and family income were assessed at first presentation (12 gestational weeks). Psychosocial risk factors including unplanned pregnancy, social support, self-esteem, marital satisfaction and work-family conflict were assessed at all three time point.

#### 2.2.1. Planned/Unplanned Pregnancy

Expectant fathers were asked to indicate whether the pregnancy was planned or unplanned.

#### 2.2.2. Social Support

Social support was measured with the multidimensional scale of perceived social support which consisted of 12 items measuring perceived social support from family, friends, and significant others [25]. Cronbach's alpha of the significant other, family and friends subscales were .91, .87, and .85, respectively. The reliability of the scale as a whole was .88 which demonstrated a good internal consistency of the scale. The test-retest reliability for the significant other, family and friends subscales were .72, .85, and .75, respectively and .85 as a whole. The validated Chinese version was used in the present study [26]. Cronbach's coefficient alphas of the MSPSS-C scale were .89 and .94 and .86 for the friend and family subscales, respectively [25].

#### 2.2.3. Self-Esteem

The 10-item Rosenberg self-esteem Scale (RSE) [27] was used to assess global self-esteem. It consists of 10 statements assessing overall feelings of self-worth and self-acceptance. The items are answered on a four-point scale ranging from strongly agree to strongly disagree. The RSE has demonstrated good reliability and validity across a large number of different sample groups and has been validated for use with both male and female adolescent, adult and elderly populations. Cronbach's alpha of the RSE is .92 [27].

#### 2.2.4. Work-Family Conflict

The work-family conflict Scale [28] is a 10-item scale developed to measure participants' perception of work-to-family and family-to-work interference. Sample items are “Due to work-related duties, I have to make changes to my plans for family activities” and “The demands of my family or partner/partner interfere with work-related activities.” Respondents were asked to respond on a 7-point scale, ranging from 1 “strongly disagree” to 7 “strongly agree”. Higher scores on the subscales represent higher levels of respective direction of work-family interference. Cronbach's alpha of the Work-Family Conflict scale ranged from .82 to .90. The scale was used to assess perceived conflicts between work and family [28].

#### 2.2.5. Marital Satisfaction

The validated 3-item Kansas Marital Satisfaction Scale [29, 30] was used to measure marital satisfaction. Cronbach's alphas are .98 and .92 for the English version and Chinese version of kansas marital satisfaction scale, respectively [29].

### 2.3. Analysis Plan

The statistical package for the social sciences (SPSS) was used for all analyses. The overall level of significance was taken as 5% and all estimates were accompanied by 95% confidence intervals. Descriptive statistics was presented by means and SDs for continuous variables and percentages for categorical variables. To examine the effect of hypothesized risk factors. ANCOVA and hierarchical hierarchical multiple regression were conducted with potential confounding factors controlled for.

## 3. Results

### 3.1. Response Rate

A total of 622 expectant fathers were recruited in the present study at 12 weeks of their partners' pregnancy. The response rate was 72.56%. At 36 weeks gestation weeks, 337 (54.2%) completed the questionnaires for both time-points, yielding an attrition rate of 45.8%. At six weeks postpartum, 150 (24.1%) participants dropped out from the study. A total of 187 (30.1%) participants completed all three time points of the survey.

### 3.2. Attrition Analyses

An attrition analysis was conducted between the group of participants who completed both antenatal time points (*n* = 337) and the group of participants who dropped out at 36 gestation weeks (*n* = 285).

Bonferroni correction was used to address the problem of multiple comparisons. The significance level of .05 was adopted in the current study. Using Bonferroni correction, each category of variables (demographic, psychosocial risk factors and main outcome variables) was tested at a significance level of .05/*n* (*n* being the number of variables which were examined in each category).

After Bonferroni correction, results showed that there was no significance difference in risk factors and main outcomes between those who completed both antenatal time points and those who dropped out at 36 weeks gestation (Table 1).

The same analysis was conducted between the group of participants who completed all time points (*n* = 187) and the group of participants who dropped out at 6 six weeks postpartum (*n* = 150).

Again, results showed that there is no significant difference in risk factors and main outcomes between those who completed all three time points and those who dropped out at six weeks postpartum (Table 2).

### 3.3. Sample Characteristics

All of the expectant fathers were recruited from 12 to 28 gestation weeks of pregnancy at their spouse's first presentation at the antenatal clinic. The average gestational week was 15.7 weeks. The majority of the first assessment (82.8%) was conducted at or before 18 weeks of gestation. The remaining minority was first assessed after 18 weeks because of late presentation at the antenatal clinic. However, Chi-square and independent *t*-tests analyses showed no differences between these two groups on core demographic variables (age: *t*  (602) = −1.67, *P* = .098 > .0036; marital status: *χ* ^2^(1, *N* = 619) = 1.46, *P* = .23; education level: *χ* ^2^(1, *N* = 591) = .72, *P* = .40; family income: *χ* ^2^(2, *N* = 602) = 6.31, *P* = .043), risk factors (unplanned pregnancy: *χ* ^2^(1, *N* = 615) = 4.13, *P* = .042; baseline marital dissatisfaction: *χ* ^2^(1, *N* = 610) = .51, *P* = .50; baseline self-esteem: *t*  (598) = 1.36, *P* = .18; baseline social support: *t*  (608) = 2.41, *P* = .016; baseline work-family conflict: *t*  (583) = −.48, *P* = .63) and main outcome variables (baseline epds score: *t*  (581) = −.094, *P* = .93); at baseline after Bonferroni correction (significance level at .05/10 = .005), hence they were treated as one group. The mean age of the expectant fathers was 34.19, and the age range was relatively large, ranging from 19 to 55 years old. Among them, 19 (3.1%) were below the age of 25 years old, 310 (51.3%) were between 26 to 34 years old, and 275 (45.5%) of them were above 35 years old.

Concerning the fathers' marital condition, most of the expectant fathers (612, 98.9%) were either married or cohabitating with their partners, and a small proportion of them (1.1%) as reported was being single or divorced.

A total of 411 (67.7%) of the expectant fathers were first-time father, and 196 (32.3%) were experienced father. Most of them (99.3%) reported no history of psychiatric illness, while 0.7% of them had a history of psychiatric illness, among them, majority suffered from depression.

Most (58.1%) of the expectant fathers were considered highly educated, having received tertiary or above education. Less than half (41%) of the expectant fathers finished secondary school education only and only .90% of them finished primary school education. Due to the small number of low educated sample, the analyses were done by combining fathers reaching primary school and secondary school education level into one group, making the percentage to 41.9%.

With regard to total family income, 58% of the families received a total monthly income of HKD 30000 or above, while 24.9% of them had a total monthly family income ranging from HKD 20000 to 30000. A small proportion of the families (17.1%) reported a relatively low family income of HKD 20000 or below per month. The summary of the sample characteristics can be viewed in Table 3.

### 3.4. Prevalence of Paternal Depression in the Antenatal and Postpartum Period

The Edinburgh postnatal depression scale (EPDS) [22] was used as a screening measure of depression of expectant fathers. EPDS consists of 10 items which assess depressive symptoms on a four-point scale (0–3), yielding a maximum score of 30. The traditional cut-off of 13 or above was used to define a probable case of depression. EPDS scores were also analyzed as continuous variable to examine the severity of depressive symptoms.

**Figure 1:**
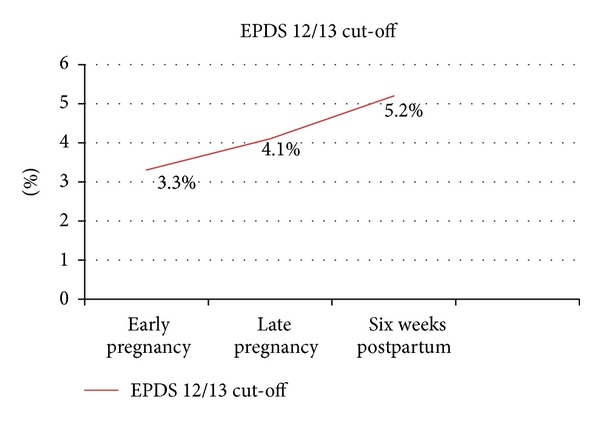
Prevalence rate of paternal depression from early pregnancy to six weeks postpartum using EPDS Scale (Recommended cut-off of ≥13).

The mean and standard deviations of the EPDS scores across early, late pregnancy and six weeks postpartum were 5.22 (SD = 3.59); 5.23 (SD = 3.67); and 5.15 (SD = 4.17) respectively. Using ≥13 as the standardized cut-off for probable case of depression, the prevalence increased as the pregnancy progress and reached a peak at 6 weeks postpartum, with 1 3.3% of the participants scoring above cut-off in early pregnancy, 4.1% in late pregnancy and 5.2% at six weeks postpartum, respectively.  Figure 1 showed the prevalence rate of paternal depression from early pregnancy to six week postpartum.

EPDS is a self-report symptoms instrument instead of a diagnostic tool to define paternal depression; hence the subsequent analyses used continuous scores of EPDS instead of cut-off scores.

### 3.5. The Relationships between Paternal Antenatal and Postpartum Depression

Using Pearson correlation, paternal depression in early pregnancy was significantly correlated with depression in late pregnancy [*r*  (299) = .53, *P* < .001] and six weeks postpartum [*r*  (195) = .55, *P* < .001]. Paternal depression in late pregnancy was also significantly correlated with paternal depression at six weeks postpartum [*r*  (167) = .57, *P* < .001] (Table 4).

To examine the independent and combined predictive power of antenatal paternal depression at both time points for postpartum paternal depression, a multiple linear regression was also performed with EPDS scores at both antenatal time points entered into the same model. It was found that both paternal depression in early pregnancy (*β* = .34, *t* = 4.74; *P* < .000) and paternal depression in late pregnancy (*β* = .39, *t* = 5.49, *P* < .000) could independently predict depression at six weeks postpartum. Combined together, the full model produced an adjusted *R* square of .39, which was statistically significant [*F*(2, 156) = 51.56, *P* < .000], indicating that antenatal paternal depression in early and late pregnancy could explain 39% of the variance in paternal postpartum depression.

### 3.6. Identification of Risk Factors

A series of statistical analyses were performed to examine which risk factors significantly predicted EPDS scores. Specifically, for dichotomous predictor variables, ANCOVA was used, with appropriate covariates entered into the model, where relevant, post hoc analyses were conducted as well. For continuous predictor variables, multiple regression analyses were conducted. Dependent variables were EPDS scores in early and late pregnancy and at 6 weeks postpartum.

#### 3.6.1. Demographic Risk Factors

Various statistical analyses were carried out to examine the relationships between demographic risk factors and expectant fathers' depression, including one-way ANOVA, Pearson's correlation and independent samples *t*-test. Post Hoc test using LSD criterion showed that fathers with low family income (mean = 5.95, SD = 4.01) had significantly higher score of EPDS compared to fathers with high family income (mean = 5.06, SD = 3.34) in early pregnancy, although the one-way ANOVA did not show significant main effect [*F*(2, 564) = 2.43, *P* = .09]. Results showed that no significant association was found between any demographic risk factors and depression in the later time points.

#### 3.6.2. Psychosocial Risk Factors

Multiple regression was conducted to test the effect of psychosocial variables on EPDS scores across different time points. In order to control for the effect of demographic variables, the significant demographic risk factor (i.e., family income in early pregnancy) was included in Block 1 as covariate. All the psychosocial risk factors were then included in Block 2 in one regression model.

In early pregnancy the following predictor variables were tested: Block 1: family income, Block 2: unplanned pregnancy, marital dissatisfaction, self-esteem, social support, and work-family conflict, with fathers' EPDS score as the outcome variable. The model produced an adjusted *R* square of .20, which was statistically significant [*F*(6, 510) = 22.42, *P* < .001]. Fathers who reported dissatisfaction with marital relationship were found to have higher scores in EPDS, (*β* = −.14, *t* = − 3.36, *P* < .001). Poor self-esteem could also predict higher scores in EPDS in fathers, with *β* = −.24, *t* = −5.37, *P* < .001. Positive association was found between work-family conflict and EPDS scores [*β* = .22, *t* = 5.24, *P* ≤ .001]. Family income, unplanned pregnancy and social support were excluded from the model.

Unplanned pregnancy, marital dissatisfaction, self-esteem, social support and work-family conflict, were entered into one regression model in order to examine the effect of psychosocial risk factors in late pregnancy. The model produced an adjusted *R* square of .26, which was statistically significant [*F*(5, 276) = 20.21, *P* < .001]. Unplanned pregnancy and marital dissatisfaction were excluded from the model. Poor self-esteem [*β* = −.28, *t* = − 4.69, *P* ≤ .001]. and poor social support [*β* = −.13, *t* = −2.21, *P* ≤ .05] could significantly predict higher EPDS scores. High levels of work-family conflict [*β* = .21, *t* = 3.75, *P* ≤ .001] were found to be significant predictors of higher EPDS scores in late pregnancy.

At six weeks postpartum, the regression model produced a good fit (adjusted *R* ^2^ = 37.3%), which was significant [*F*(5, 185) = 23.00, *P* < .001]. Poor self-esteem [*β* = −.45, *t* = −6.61, *P* ≤ .001] and work-family conflict [*β* = .18, *t* = 2.68, *P* ≤ .008] at six weeks postpartum were found to be strong predictors for higher EPDS scores among the expectant fathers. The other predictors were not significant.

The results of multivariate analyses are summarized in Tables 5, 6, and 7.

## 4. Discussion

The large scale survey by National Institute of Mental Health (NIMH) in 2005 documented the 12 months prevalence of major depressive disorder among United States' male adults to be 5.2% using DSM-IV [31]. The results of the present study showed that the prevalence rates of probable and significant cases of depression among expectant fathers elevated across early and late stages of pregnancy and reached the peak at 6 weeks postpartum, increasing from 3.30% to 4.1% to 5.20% for significant cases. Previous studies in the literature on paternal postpartum depression reported a wide range prevalence rate, ranging from 5.3 to 31.7% for paternal postpartum depression [7–9, 32]. Compared to the maternal perinatal depression rates which range from 7 to 20% [1–5, 33–35], the prevalence of paternal perinatal depression was not low and should not be overlooked. A few previous studies had used EPDS to assess the psychological distress in fathers. Ballard et al. [7] assessed both members of 150 couples at 6 weeks and 6 months postnatally using the Edinburgh Postnatal Depression Scale (EPDS) [22] with a cut-off score of 12/13. The prevalence of paternal depression at 6 weeks and 6 months postpartum was found to be 9% and 5.4% respectively [7]. Deater-Deckard et al. [36] assessed 7018 partners of a representative British women in a longitudinal study using the EPDS showed that 3.5% and 3.3% of men were above cut-off of 13 at 18 weeks gestation and 8 weeks postpartum respectively [36]. It is rather difficult to draw a general consensus on the prevalence rate of paternal depressive symptoms as the timing of assessments differ widely and the study populations are not comparable. However, a few pioneer longitudinal study regarding expectant fathers' depression [14, 17, 21] stated that expectant fathers demonstrated more symptoms of distress, including becoming more depressed and irritable as well as having more negative effect in the postnatal period. Our study shed light on the prevalence of depressive symptoms among the population of Chinese expectant fathers and is in line with the studies above, suggesting that expectant fathers in Hong Kong is more susceptible to depressive symptoms in the postpartum period.

Studies on the relationships of maternal antenatal and postpartum depression showed that maternal depression during pregnancy was the strongest predictor of maternal postpartum depression [3, 15, 16]. In the present study, we found that paternal depression in early pregnancy, late pregnancy, and six weeks postpartum were strongly correlated with each other. We further found that paternal antenatal depression, especially paternal depression in late pregnancy, could significantly predict higher level of depression among the expectant fathers in the postpartum period. This indicates that paternal depression screening and interventions should be done as early as in early pregnancy in order to prevent the high risk expectant fathers from developing depression in the postpartum period which can lead to detrimental effect on their spouse and children's development.

Preliminary studies showed that the risk factors associated with paternal perinatal psychological distress and mental health problems included poor marital relationship, poor social network and insufficient information about pregnancy and childbirth, having an unsupportive relationship, past history of psychiatric disorder, young age, being unemployed and poor social function [17–20]. Poor marital satisfaction, low social support and poor quality of life were found to be associated with paternal postpartum depression [17].

The current findings not only confirmed the salience of psychosocial factors identified in previous studies as strong predictors of paternal depression across the perinatal period but also contribute to new knowledge by identifying additional risk factors. Poor self-esteem, poor social support, marital dissatisfaction, high level of work-family conflict were found to significantly predict higher level of EPDS scores in fathers in different time points during the perinatal period. Cronenwett and Kunst-Wilson [37] stated that men tended to have poorer social support networks compared to women as men tend to rely primarily on their partners for support after getting married [37]. A previous study which was done on the expectant fathers found that the transition from a dyadic to a triadic relationship during the perinatal period was a threat to the men especially for those with poorer social support systems outside the marital relationship in the perinatal period [17]. These results are consistent with our current findings with marital dissatisfaction predicting higher EPDS score in early pregnancy and poor social support predicting higher EPDS score in late pregnancy and six weeks postpartum. Paternal stress is compounded by the fact that resources for parenting are mostly maternal-oriented. As many as 40% of men stated that they often felt that the information available for them related to pregnancy and childbirth is limited, and that they required more information to prepare them for their role and tasks as fathers (BBC News report, 2000). Many of the expectant fathers also had a lack of good role model of fathering for they were being brought up in times when men were less involved in birth and child-rearing. These factors weakened the confidence of the expectant fathers and left them wondering if they would be competent in childrearing. In fact, a study by Ferketich and Mercer [38] clearly stated that in early postnatal months, the fathers “paternal role competence” was strongly predicted by the men's sense of mastery and their perception of family functioning. Our current results echoed with this study's results and suggested that poor self-esteem and work-family conflict led the men with a negative sense of mastery or control to be at risk of failing to achieve the “competent paternal role” which might contribute to the higher score of EPDS in the early postpartum period [38]. And High level of work-family conflict which was not reported in previous literature was a strong predictor of paternal mental health problems across different time points. Work-family balance was found to be notoriously poor in the families in Hong Kong where the demands of working hours and quality are extremely high. A study on a sample of Hong Kong Chinese employed parents in dual-earner families indicated that work-family conflict was negatively related to job and life satisfaction. The study also showed that the coping behaviors of Hong Kong employed parents were largely ineffective against the problem of work-family conflict [39]. The current study shed light on how work-family conflict had an impact on expectant couples' mental health and hopefully raise the awareness of the public to pay attention to the importance of maintaining work life balance. Interventions on helping the fathers to cope with poor self-esteem, poor social support, marital dissatisfaction and work-family conflict could be very beneficial.

Acknowledgment of the limitations of the present study is essential to make appropriate interpretation and generalization of the results. It also guides the directions for future research. First, due to time constraint and scope of the present study, we could only study depressive symptoms of the fathers until six weeks postpartum. Six weeks postpartum might be a period which was still quite hectic for new parents as they just entered the new life of parenthood and were still pretty green with taking care of the babies. This might increase the risk of overestimating the prevalence rate of paternal depression. Also, the effect of the maternal depression on expectant fathers was well addressed to be a significant predictor of paternal depression in previous studies [9, 32]. In future studies, maternal depression should be included as a risk factor and possibly as a potential confounder as well in order to further clarify the relationships between other risk factors and paternal depression. It should also be emphasized that the EPDS is a screening instrument and not a diagnostic tool. The rates detected only indicated probable depression. Future studies should consider including a diagnostic interview to confirm the clinical status of the fathers' depression. It should also be acknowledged that the data was collected from the antenatal clinic in one district in Hong Kong only. The sampling did not include subjects from the other public or private clinics or hospitals in other districts in Hong Kong. Indeed, it consisted of a relatively highly-educated group with high income and low reported rates of psychiatric disorders. Caution should be exercised in generalizing the results to fathers of other backgrounds. In all likelihood, the current finding is an underestimate of the prevalence and severity of paternal depression.

With time, the severity of paternal depression might be reduced as fathers develop more adaptive coping. Thus, the current design might not be able to capture full picture of paternal postpartum depression. Another potential problem is that the fathers who had nonpregnant partners were not included in the present study as the comparison group. Although the prevalence rate of depression in the healthy control group of men from National Institute of Mental Health was included in discussion, it could not give a clear picture on comparisons as the DSM-IV was used to define the cases of depression in the NIMH study. Matched controls should be included in future studies in order to provide a reliable comparison group for the purpose of investigating prevalence rate as well as identifying risk factors. In addition, the present study used self-report symptoms instruments instead of diagnostic tools to define paternal mental health problems. As stated in the literature, males tended to hide the emotions they experienced in comparison to females which justified the under estimation of the rate of males' mental health problems [40–42]. To minimize the bias, continuous scores for the scales were used in the analyses instead of cut-off scores. To tackle the problems, it might be helpful to include the diagnostic tools into the study for the future prospect.

## 5. Conclusion

In conclusion, expectant fathers in Hong Kong scored higher in EPDS in the postpartum period compared to the antenatal period and paternal depression during the perinatal period should not be overlooked. As maternal perinatal depression is being documented as a significant health problem, it should be acknowledged that fathers, being one of the parents, also experience a phase of transition and substantial stress during and after their partners' pregnancy. Thus, prevention, early identification and intervention of paternal perinatal depression are needed.

The present study has also investigated the relationship between paternal antenatal and postpartum depression as well as identified significant demographic and psychosocial risk factors for paternal perinatal depression. Such knowledge contributes to the effective design of screening, prevention, and intervention strategies and also helps in the identification of high risk groups.

## Figures and Tables

**Table 1:** Sample characteristics and attrition analysis of expectant fathers with comparison between fathers who completed both antenatal time points and fathers who dropped out in late pregnancy.

	Participants who dropped out at 36 weeks of gestation (*n* = 285)		Participant who completed both antenatal time points (*n* = 337)		
Significance level at Bonferroni correction: 0.05/5 = 0.01					

Demographic risk factors					

	Mean	SD	Mean	SD	

Age	(18)				
	33.86	5.04	34.47	5.34	*t* (602) = −1.45 *P* = .15

	*N*	%	*N*	%	

Marital status	(3)				
Married/cohabitating	281	45.18	331	53.22	*χ* ^2^ (1, *N* = 619) = .026, *P* = .87
Divorce/single	3	0.48	4	0.64	
Parity	(15)				
Primigravida	191	30.71	220	35.37	*χ* ^2^ (1, *N* = 607) = .52, *P* = .47
Multigravida	85	13.67	111	17.85	
Education level	(2)				
Secondary or below	128	20.58	132	21.22	*χ* ^2^ (1, *N* = 620) = 2.12, *P* = .15
Tertiary or above	156	25.08	204	32.80	
Family income	(20)				
<20000	46	7.40	57	9.16	*χ* ^2^ (2, *N* = 602) = .58, *P* = .75
20000–30000	73	11.74	77	12.38	
>30000	158	25.40	191	30.71	

Significance level at Bonferroni correction: 0.05/5 = 0.01					

Psychosocial risk factors					

	*N*	%	*N*	%	

Planned/unplanned pregnancy	(7)				
Planned pregnancy	219	35.21	276	44.37	*χ* ^2^ (1, *N* = 615) = 1.69, *P* = .19
Unplanned pregnancy	61	9.81	59	9.49	
Marital dissatisfaction	(12)				
Marital distress	42	6.75	45	7.23	*χ* ^2^ (1, *N* = 610) = .34, *P* = .56
Marital satisfied	235	37.78	288	46.30	

	Mean	SD	Mean	SD	

Self-esteem	(24)				
	20.71	4.60	21.43	4.50	*t* (598) = −1.95 *P* = .052
Social-support	(14)				
	61.77	14.77	63.48	13.65	*t* (608) = −1.49, *P* = .14
Work-family conflict	(39)				
	30.63	11.90	30.36	11.70	*t* (583) = −1.95, *P* = .052

Significance level at Bonferroni correction: 0.05/7 = 0.007					

Baseline main outcome variables					

Mental health problems	Mean	SD	Mean	SD	

Depression	(41)				
	5.32	3.59	5.12	3.59	*t* (581) = .67, *P* = .50

**Table 2:** Sample characteristics and attrition analysis of expectant fathers with comparison between fathers who completed all time points and fathers who dropped out at six weeks postpartum.

	Participants that dropped out at 6 weeks postpartum (*n* = 150)		Participant that completed all 3 time points (*n* = 187)		
Significance level at Bonferroni correction: 0.05/5 = 0.01					

Demographic risk factors					

	Mean	SD	Mean	SD	

Age	(13)				
	33.95	4.94	34.88	5.61	*t* (322) = −1.56 *P* = .12

	*N*	%	*N*	%	

Marital status	(2)				
Married/cohabitating	146	43.32	185	53.22	*χ* ^2^ (1, *N* = 335) = 1.53, *P* = .22
Divorce/single	3	0.89	1	0.30	
Parity	(6)				
Primigravida	101	29.97	119	35.31	*χ* ^2^ (1, *N* = 331) = .38, *P* = .54
Multigravida	47	13.95	64	18.99	
Education level	(1)				
Secondary or below	59	17.51	73	21.66	*χ* ^2^ (1, *N* = 336) = .011, *P* = .92
Tertiary or above	90	26.71	114	33.83	
Family income	(12)				
<20000	21	6.23	36	10.68	*χ* ^2^ (2, *N* = 325) = 3.67, *P* = .16
20000–30000	30	8.90	47	13.95	
>30000	93	27.60	98	29.08	

Significance level with Bonferroni correction: 0.05/9 = 0.0056					

Psychosocial risk factors					

	*N*	%	*N*	%	

Planned/unplanned pregnancy	(2)				
Planned pregnancy	115	34.12	161	47.77	*χ* ^2^ (1, *N* = 335) = 5.01, *P* = .025
Unplanned pregnancy	34	10.10	25	7.42	
Baseline marital dissatisfaction	(4)				
Marital distress	17	5.04	28	8.31	*χ* ^2^ (1, *N* = 333) = .86, *P* = .36
Marital satisfied	130	38.58	158	46.88	
Marital dissatisfaction in late pregnancy	(13)				
Marital distress	18	5.34	29	8.61	*χ* ^2^ (1, *N* = 324) = .84, *P* = .36
Marital satisfied	126	37.39	151	44.81	

	Mean	SD	Mean	SD	

Baseline self-esteem	(14)				
	21.49	4.68	21.39	4.36	*t* (322) = −1.56 *P* = .12
Self-esteem in late pregnancy	(8)				
	21.67	4.53	21.44	4.36	*t* (327) = .47 *P* = .64
Baseline social support	(8)				
	64.82	13.08	62.42	14.03	*t* (327) = 1.59, *P* = .11
Social support in late pregnancy	(5)				
	63.76	13.45	61.36	14.78	*t* (330) = 1.52, *P* = .13
Baseline work-family conflict	(21)				
	30.52	12.02	30.24	11.47	*t* (314) = .21, *P* = .83
Work-family conflict in late pregnancy	(25)				
	31.22	12.04	30.88	12.96	*t* (310) = .24, *P* = .81

Significance level with Bonferroni correction: 0.05/13 = 0.0038					

Main outcome variables					

Mental health problems	Mean	SD	Mean	SD	

Baseline depression	(22)				
	5.00	3.59	5.22	3.60	*t* (313) = −.55, *P* = .58
Depression in late pregnancy	(19)				
	5.31	4.07	5.17	3.32	*t* (316) = .36, *P* = .72

**Table 3:** Sample characteristics of expectant fathers in the present study.

Characteristic	*n*	%
Age	(18)	
≤25	19	3.1
26–34	310	51.3
≥35	275	45.5
	Mean age = 34.19, SD = 5.21	
Marital status	(3)	
Married/cohabitating	612	98.9
Single/divorced	7	1.1
History of psychiatric illness	(9)	
Yes	4	.7
No	609	99.3
Parity	(15)	
Primigravida	411	67.7
Multigravida	196	32.3
Education Level	(2)	
Secondary	260	41.9
Tertiary or above	360	58.1
Family income	(20)	
<20000	103	17.1
20000–30000	150	24.9
>30000	349	58.0

**Table 4:** Correlation of paternal depression from early pregnancy to six weeks postpartum.

		Depression in early pregnancy	Depression in late pregnancy	Depression at six weeks postpartum
Depression in early pregnancy	*r*		.53**	.55**
Depression in late pregnancy	*r*	.53**		.57**
Depression at six weeks postpartum	*r*	.55**	.57**	

**Correlation is significant at 0.001 level (2 tailed).

*Correlation is significant at 0.05 level (2 tailed).

**Table 5:** Hierarchical multiple regression analysis of significant psychosocial risk factors for EPDS scores in early pregnancy.

Model	Early pregnancy				
	Edinburgh postnatal depression scale (EPDS)				
	Adjusted *R* ^2^	*F-*value	*β*	*t*	*P*
1	.011	5.47			.020*
Family income			−.06	−1.47	.14
2	.20	22.42			.000**
Unplanned pregnancy			.016	.40	.69
Marital Satisfaction			−.14	−3.36	.001**
Self-esteem			−.24	−5.37	.000**
Social-support			−.039	−.92	.36
Work-family conflict			.22	5.24	.000**

**Table 6:** Hierarchical multiple regression analysis of significant psychosocial risk factors for EPDS scores in late pregnancy.

	Late pregnancy				
	Edinburgh postnatal depression scale (EPDS)				
	Adjusted *R* ^2^	*F-*value	*β*	*t*	*P*
	.27	20.21			.000**
Unplanned pregnancy			.096	1.82	.069
Marital satisfaction			−.092	−1.69	.092
Self-esteem			−.28	−4.69	.000**
Social-support			−.13	−2.21	.028*
Work-family conflict			.21	3.75	.000**

**Table 7:** Hierarchical multiple regression analysis of significant psychosocial risk factors for EPDS scores at six weeks postpartum.

Model	Six weeks postpartum				
	Edinburgh postnatal depression scale (EPDS)				
	Adjusted *R* ^2^	*F-*value	*β*	*t*	*P*
	.39	23.00			.000**
Unplanned pregnancy			−.019	−.32	.75
Marital satisfaction			−.048	−.78	.44
Self-esteem			−.45	−6.61	.000**
Social-support			−.12	−1.84	.068
Work-family conflict			.18	2.68	.008*
